# Reproductive biology of the encapsulating, brooding gastropod *Crepipatella dilatata* Lamarck (Gastropoda, Calyptraeidae)

**DOI:** 10.1371/journal.pone.0220051

**Published:** 2019-07-23

**Authors:** Oscar R. Chaparro, Víctor M. Cubillos, Jaime A. Montory, Jorge M. Navarro, Paola V. Andrade-Villagrán

**Affiliations:** 1 Instituto de Ciencias Marinas y Limnológicas, Universidad Austral de Chile, Valdivia, Chile; 2 Laboratorio de Recursos Acuáticos y Costeros de Calfuco, Universidad Austral de Chile, Valdivia, Chile; 3 Centro i~mar, Universidad de Los Lagos, Puerto Montt, Chile; 4 Centro Fondap de Investigación Dinámica de Ecosistemas Marinos de Altas Latitudes (IDEAL), Universidad Austral de Chile, Valdivia, Chile; 5 Centro de Investigación en Biodiversidad y Ambientes Sustentables (CIBAS), Facultad de Ciencias, Universidad Católica de la Santísima Concepción, Concepción, Chile; Gettysburg College, UNITED STATES

## Abstract

Among calyptraeid gastropods, males become females as they get older, and egg capsules containing developing embryos are maintained beneath the mother’s shell until the encapsulated embryos hatch. *Crepipatella dilatata* is an interesting biological model considering that is an estuarine species and thus periodically exposed to elevated environment-physiological pressures. Presently, there is not much information about the reproductive biology and brooding parameters of this gastropod. This paper describes field and laboratory observations monitoring sex changes, brooding frequencies, sizes of brooding females, egg mass characteristics, and embryonic hatching conditions. Our findings indicate that *C*. *dilatata* is a direct-developing protandric hermaphrodite, changing from male to female when individuals were between 18 and 20 mm in shell length. At our study site in Quempillén estuary, females were found to be brooding almost continuously throughout the year, having an average maximum of 85% of simultaneous brooding, with a short rest from April through June. No relationship was found between the number of capsules per egg mass and the size of the brooding female. However, capsule size and the number of embryos and nurse eggs were strongly related to female size. The offspring hatched with an average shell length > 1 mm. About 25% of the hatched capsules were found to contain both metamorphosed (juveniles) and non-metamorphosed (veliger) individuals. The sizes of the latter were < 1000 μm. The length of hatching juveniles was inversely related to the number of individuals per capsule, which seems related to differences in the availability of nurse eggs per embryo. Although fecundity per reproductive event of this species is relatively low (maximum approx. 800 offspring per egg mass) compared with those of calyptraeid species showing mixed development, the overall reproductive potential of *C*. *dilatata* seems to be high considering that females can reproduce up to 5 times per year, protecting their encapsulated embryos from physical stresses until well-developed juveniles are released into the population, avoiding a dangerous pelagic period prior to metamorphosis.

## Introduction

Benthic marine invertebrates present a wide variety of reproductive patterns in their life histories [1,2]. Some have external fertilization, through the release of gametes into the water column, where a free-swimming larva is generated, continuing its development until settlement and metamorphosis take place [3,4]. In these cases, there is no maternal care of their offspring. In contrast, in some other species (e.g. prosobranchs), fertilization occurs internally and mothers then enclose groups of embryos within mucus, egg-masses, or egg capsules. Although in some species, females produce egg capsules and then leave them alone, others brood their fertilized eggs until the embryos hatch, generating a close physical relationship between the mother and her offspring [5,6]. Although, through incubation, the capsules are physically protected by the mother [7–9], their multi-layered walls provide some extra levels of protection to the initial stages of development [2,10–13].

The species of the gastropod family Calyptraeidae (Caenogastropoda) are protandrous hermaphrodites, and incubate encapsulated embryos for different periods of time [14,15]. The egg capsules are laid on the substrate and protected beneath the mother’s shell during embryonic development [3,15]. Previous studies indicate that encapsulation can generate benefits to embryos facing unfavorable environmental conditions [5,16,17], minimizing mortality levels due to the reduction or elimination of the pelagic phase, which is considered high risk [18–20], as well as offering a physical barrier against predators [21].

Among calyptraeids, species with “mixed development” [11] are characterized by brooding embryos inside leathery capsules, usually without extra-embryonic food, until the they hatch as veliger larvae, which then remain in the water column until settlement and metamorphosis occurs [3,4,15,22–24]. In contrast, in “direct development,” individuals hatch from the capsules as metamorphosed juveniles, often after the veligers have ingested nutritional eggs while inside the egg capsules [3,4,15,22,25–27]. The availability and intake of extraembryonic food material can influence the size at which encapsulated embryos hatch [12,28,29].

In Chile, one of the most abundant calyptraeid gastropods inhabiting the intertidal and shallow subtidal coastal areas is the direct-developing species *Crepipatella dilatata* (previously identified as *Crepidula dilatata*, see Collin 2003b [30]). This species is distributed from southern Peru, throughout the Chilean coast and part of the southern coast of Argentina [27]. In Chile, its distribution has been associated with coastal marine environments [22]. In some cases, abundant populations of this species have been identified developing in strongly estuarine environments [31]. The mode of reproduction, including maternal physical care and direct development, seem to explain the success of the development of these populations in estuarine areas, which by definition are characterized with high fluctuations in environmental variables, particularly in salinity levels [5,32,33].

Previous studies on this species have focused mainly on morphology [22,26], behavior [34], feeding mechanisms [26,35–37], physiological responses [6,38–41], and, to a lesser extent, on reproductive-energy issues [25,29,42]. According to the high density of some of its populations and the obvious ecological role playing by this species in estuarine areas, the knowledge of the reproductive biology of the species appears as an urgent need that has not been addressed in a comprehensive manner. This is particularly interesting, considering that although at mid-latitude areas most invertebrates have very marked seasonal cycles of reproduction, and essentially restricted to spring-summer (*e*.*g*. January-March *Mytilus chilensis* [43], October-December, *Ostrea chilensis* [44]), while *C*. *dilatata* deviates from this pattern, reproducing and brooding embryos during most of the year [25]. Presently, most investigations carried out with *C*. *dilatata* have considered physiological responses of adults, juveniles and encapsulates embryos under field and laboratory conditions in order to understand how salinity changes, as a consequence of tidal fluctuation, influence behavioral, metabolic and cellular responses of this euryhaline limpet in a mid-latitude estuary of the Southern hemisphere [6,33]. Although several investigations have been published concerning this biological model, little is known about its reproductive biology and brooding parameters.

Studies on population biology, genetic structure, physiology, and ecology of a species require information on reproductive biology. The present research therefore was designed to characterize the reproductive process of *C*. *dilatata* in a comprehensive manner through the study of a population of this gastropod that inhabits estuarine areas in southern Chile, where salinity conditions are highly fluctuating [32].

## Material and methods

### Brooding cycles

Females of *C*. *dilatata* were collected monthly during the periods 2003–2004 and 2006–2007 from a shallow subtidal population of the Quempillén River estuary (41 ° 52'S, 73 ° 46'W, Chiloé Island, Southern Chile) to estimate the percentage of females brooding in the population at different times of year. For this study, we considered only those individuals with a shell length that was at least as large as that of the smallest brooding female found during each sampling period (≥18 mm shell length). Water temperatures and salinities through an annual sampling period were recorded in the estuary only for the 2006–2007 sampling period, using a CTD installed 5 cm above the bottom of the estuary, where abundant specimens of *C*. *dilatata* are found.

### Shell length in brooding and non-brooding females. Change of sex from male to female

Females collected from 2003 to 2004, were detached from their substrates, and the presence or absence of egg masses was recorded. Simultaneously, the shell length (longest axis) was measured from each female to the nearest 0.01 mm using calipers.

To compare the tissue content of brooding and non-brooding females, we measured (during October samplings) the female shell lengths and then extracted the soft tissues of the specimens and place them on pre-weighed, numbered pieces of aluminum foil. The dry tissue weight (DW) was later determined by weighing the foil after the samples were kept in a drying oven for 24–48 h at 75 °C.

Considering the protandric hermaphroditism of this species, the size at which individuals transformed to females in this population was also identified, through histological analysis of a set of specimens of different sizes (N = 29), obtained from limpets collected in the estuary. The minimum and maximum sizes of female specimens were identified by the presence of brooded egg masses under its shell. Gonadal histological analyzes were performed on specimens taken directly from the estuary in November 2003. The histology was carried out using the classical techniques of fixation, dehydration, assembly and staining of the histological sections [45]. The presence of female and/or male gametes was determined from histological sections of the gonads.

### Annual spawning frequency in marked females

During September 2007, specimens > 25 mm in shell length were detached from substrates in the lab. Females were then kept on transparent glass plates in aquaria in the laboratory, to allow them to re-adhere to the new substrate. Those reattached females were marked with a number on the shell in order to make a temporal follow-up of each individual. After several days all attached females were returned to the place from where they had been collected in the estuary. Marked females remained in the estuary for two months before we began examining them each week for the presence of egg masses over the next 12 months. The presence or absence of egg masses brooded by the experimental females was easily determined by viewing through the transparent substrate to which the snails were attached. The developmental stages characterizing each of the brooded egg mass were categorized considering their coloration as follows: yellow capsules corresponded to recently laid egg masses, beige/reddish colour represent egg masses with intermediate stages of development (veliger), while egg masses containing the most advanced stage of development (juvenile) were represented by those in a dark brown color. Using this scale, it was possible to establish the number of times that a female was able to generate egg masses during the one year of observation. In this experiment, the sperm supply was not considered a limiting factor, due to the constant presence of mobile males among the controlled sessile females.

### Encapsulation and embryo hatching characteristics

Egg masses from brooding females were collected monthly during 2003–2004, placed in Eppendorff tube, and fixed with 70% alcohol in order to estimate the following parameters: total number of capsules, developmental stage of encapsulated embryos, and embryonic shell length. In the middle of each sampling season (e.g. January, March, July and September) we recorded extra information on the reproductive variables; in addition to the measurements mentioned above, we also measured capsule heights (excluding the fixation peduncle) and the maximum width in the apical region for between 20 and 50% of the capsules from each brooding female [29]. Additionally, in those capsules in which it was possible (identifiable embryos but in a stage prior to the ingestion of nurse eggs), the number and size of embryos and the number of nurse eggs present inside capsules were also quantified. In the egg masses, levels of embryonic development were recorded to identify, massive spawning events in the population. Masses containing egg-onset cell division and onset of velum formation were considered to have been recently deposited by the mother.

### Characteristics of individuals at the time of hatching

Physical characteristics of the offspring at the time of hatching were recorded in the laboratory. During spring of 2013, we collected 51 egg masses from females brooding advanced capsules in the field, and from them we used a total of 293 egg capsules for the following experiments.

Egg capsules from each spawning mass was divided through the attaching base avoiding damage to the surrounding structure, and were kept individually in small containers (10 ml volume), which were filled with filtered (0.45 um) and sterilized (UVR) seawater at 15°C and a salinity of 30 psu. The seawater was intensively air bubbled before filling the containers. Water was daily exchanged. Egg capsules (n = 293) were monitored twice a day, to identify the hatching embryos. From each recently hatched egg capsule, the number of veliger larvae and juvenile was quantified. Additionally, the length of each individual’s shell (juveniles and veligers) was recorded.

### Statistical analysis

The normality and variance homoscedasticity were corroborated using Shapiro-Wilk test and Levene test, respectively [46]. Subsequently, the comparison of the tissue content between brooders and non-brooding individuals of *C*. *dilatata* was made by means of an ANCOVA, using as individual shell length as a covariate. The same analysis was used to compare the height and width of the capsules incubated in the different seasons of the year. Analysis of ANCOVA allowed us to identify possible differences between the seasons of the year in fecundity, number of embryos per capsule, and number of total eggs deposited in an egg mass and inside a single capsule. In all comparisons, female shell length was used as the covariate. In all analyses a significance level of 0.05 was used to determine if the differences were statistically different [46].

### Ethics statement

The species involved in this research is not endangered or protected, so that no specific permissions are required to sample or remove individuals from this location.

## Results

### Brooding cycles and environmental temperature

**Fig 1:**
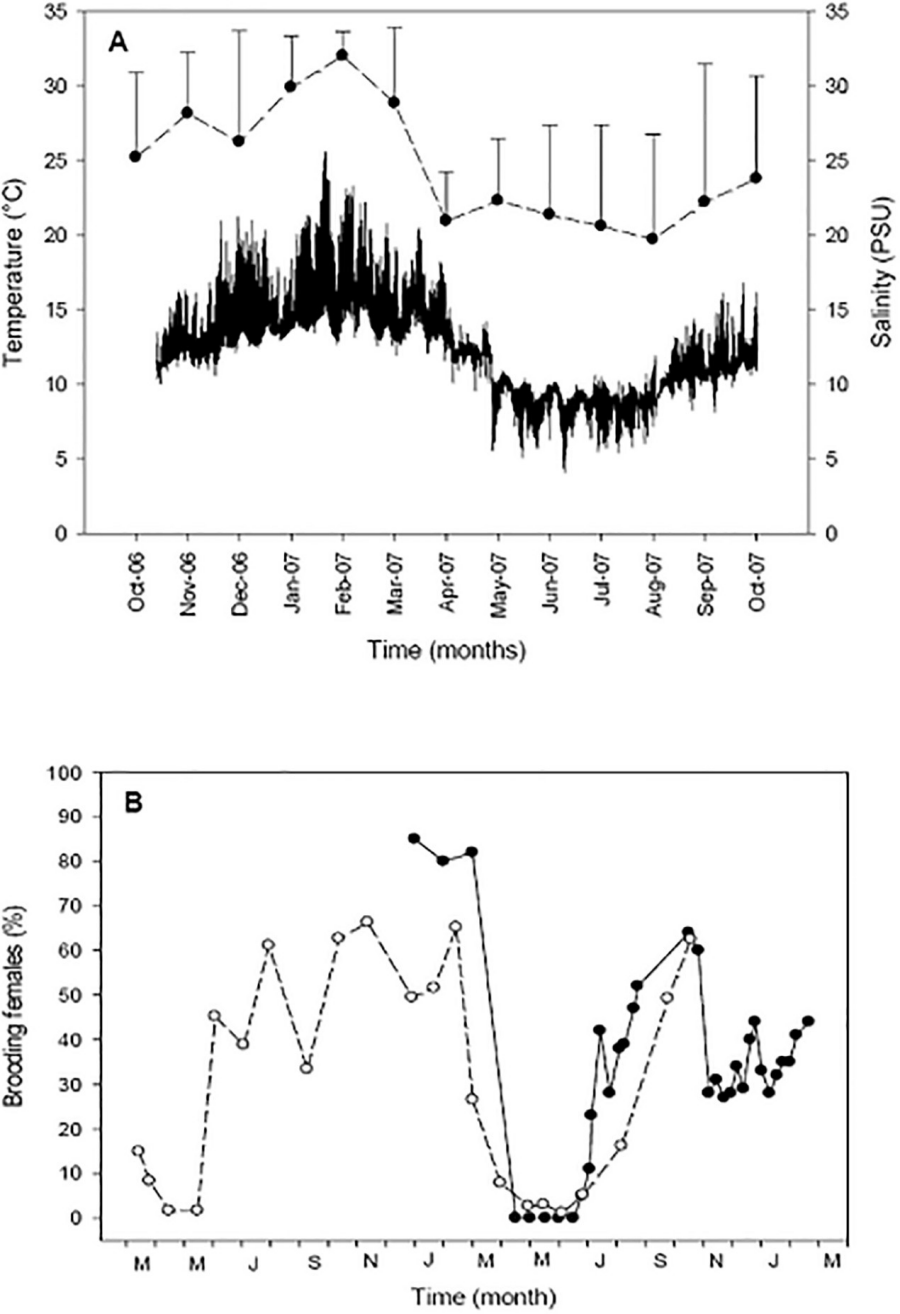
Crepipatella dilatata. Fluctuations of the A) temperature and salinity (monthly mean ± SD) of the estuary’s water during the period 2006–2007; B) brooding cycles of *C*. *dilatata* in the Quempillén estuary during 2003–2004 (empty circles) and 2006–2007 (filled circles).

The environmental temperature cycles showed strong seasonal and daily changes. The highest temperature in the estuary were recorded during spring and summer (26 °C), while the lowest value was recorded in autumn and winter (5 °C) (Fig 1A). Mean salinities in the estuary followed the temperature cycle, with the highest mean values during summer season. Brooding females were found during most of the year. The periods of greatest reproductive intensity were recorded during spring and summer, with values ranging between 65% to 85% of the females in simultaneous brooding process (Fig 1B). Only during the winter period were few females (only 2 to 3% of the potentially reproductive females) found to be brooding egg capsules (Fig 1B).

### Shell length of brooding and non-brooding females, and sizes at sex change from male to female

**Fig 2:**
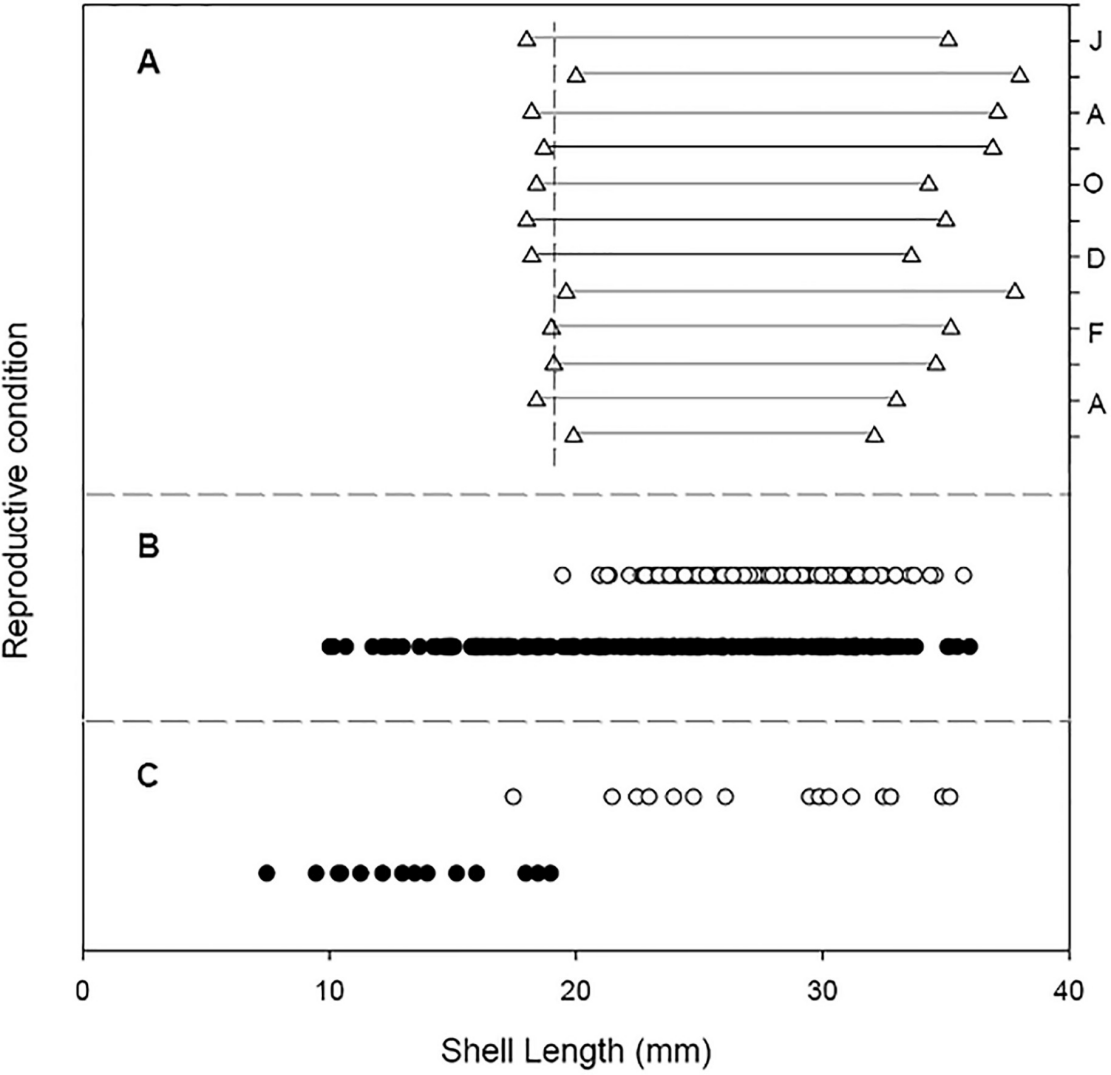
Crepipatella dilatata. Reproductive condition and size of *C*. *dilatata* specimens sampled in the Quempillén estuary. A: 2003–2004 extreme ranges of size of the brooding females according to the sampling months. B: Brooding (open circles) and non-brooding (filled circles) condition of the specimens sampled during November 2003. C: Gonadal histology conditions (open circles: females, filled circles: males) and their relation to the shell length of individuals sampled during the month of November 2003.

In general, the minimum shell length of the brooding females was 18 mm (Fig 2A and 2B). On the other hand, the biggest females found brooding had shell lengths of up to 38 mm (Fig 2A and 2B). Histological studies of the gonad (Fig 2C) corroborate that individuals change sex from male to female occurs when limpets have a shell length between 18 and 20 mm.

### Annual reproductive capacity in the estuary, based on marked specimens in the population

**Fig 3:**
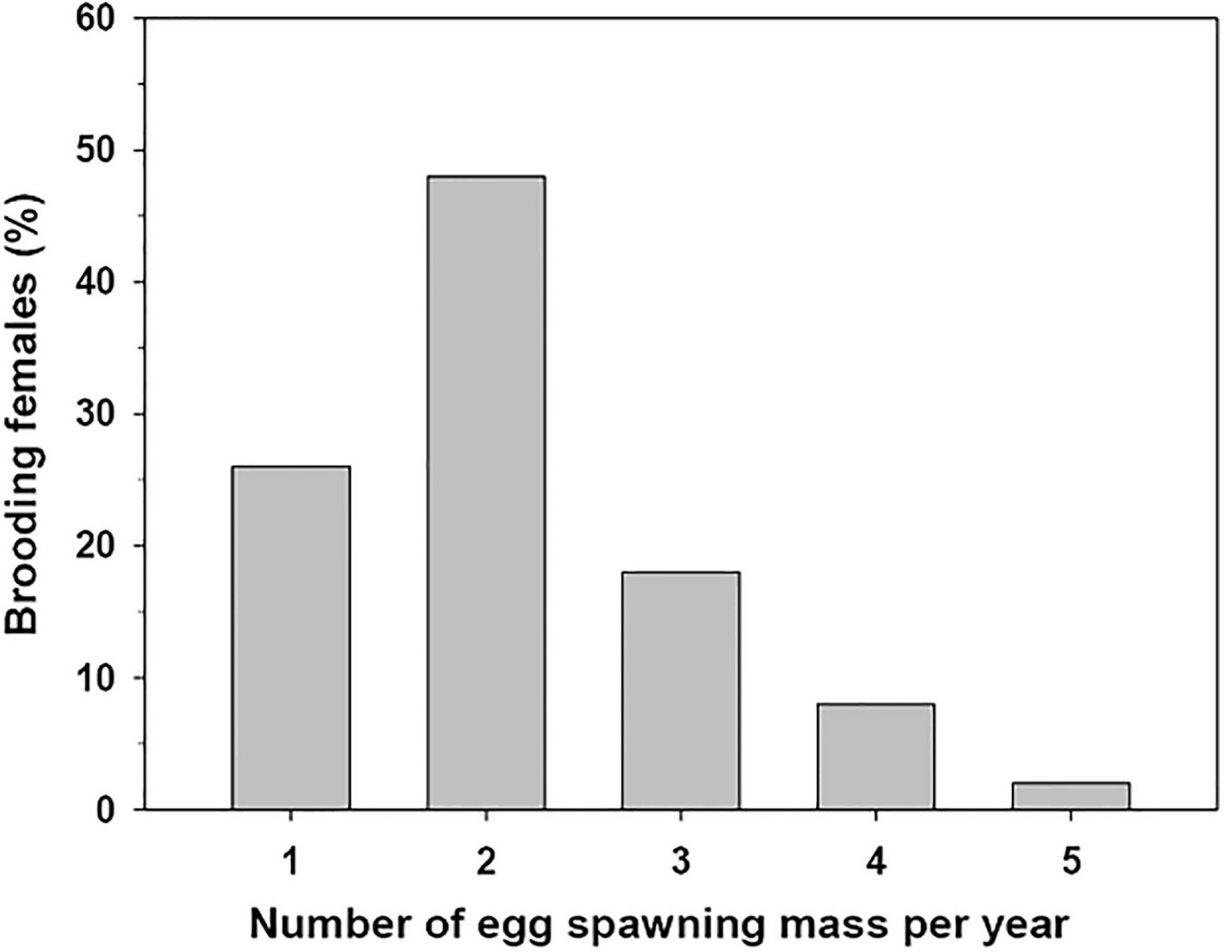
Crepipatella dilatata. Frequency of brooding in adult *C*. *dilatata* kept in the estuary and inspected every 7 days for a total period of 12 months. The assessment was made by direct observation of the brooding area in the females, which were attached to transparent substrates. The coloration gave an approximation of the developmental stage of the observed egg masses (yellow capsules: freshly laid, reddish capsules and light brown: intermediate advance level, intense brown coloration: close to hatching).

During 2007, the weekly observations performed on females tagged and maintained in the estuary, showed that the some specimens can brood up to 5 times a year (Fig 3). Notwithstanding, the most frequent number of brooding per female in the population corresponded to 2 brooding events per year and this was identified in approx. 50% of the females controlled during the experiment.

**Fig 4:**
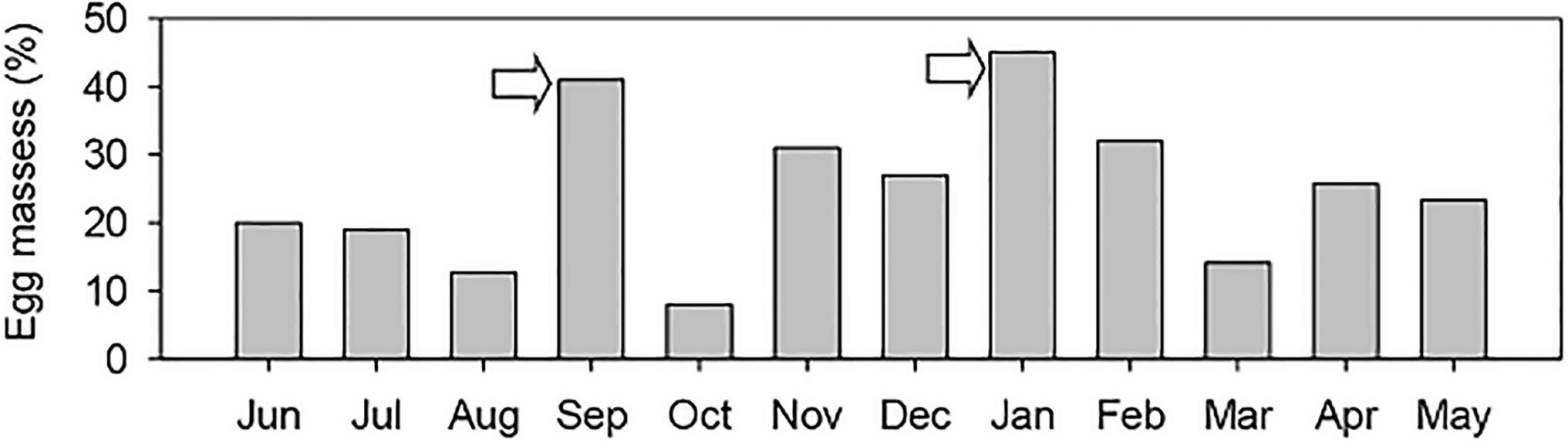
Crepipatella dilatata. Percentage of monthly egg masses of *C*. *dilatata* in the initial stage of development during the one year of sampling (2003–2004). Arrows indicate the periods with the highest proportion of egg masses containing very early developmental stages from the population of Quempillén.

At the population level, and through the identification of recent laid egg masses, it was possible to identify 2 massive spawning events, circumscribed at the beginning of spring and mid-summer (Fig 4).

### Female characteristics and their relationship to brooding

**Fig 5:**
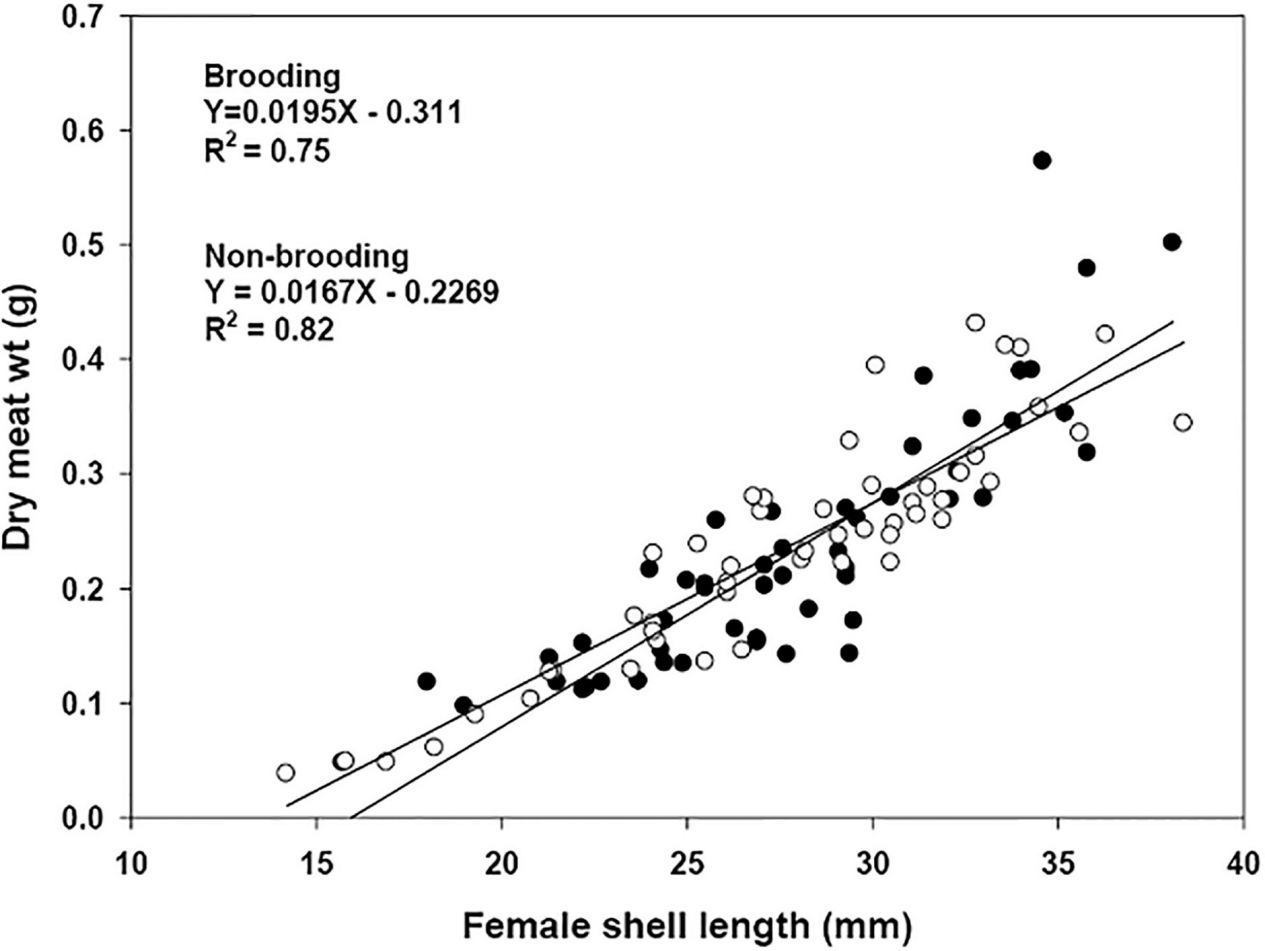
Crepipatella dilatata. Relationship between the length of the shell and meat content (dry meat weight, DMW) for brooding (including embryos at all levels of development, N = 51) and non-brooding (N = 50) females of *C*. *dilatata*. Samples were assessed in the spring season (October 2004).

There were no significant differences in dry soft tissue content of brooding and non-brooding females of *C*. *dilatata* (ANCOVA, F_(1,98)_: 0.3901, p = 0.533). The minimum and maximum levels of dry tissue weight recorded were 0.05 g and 0.57 g for individuals with a shell length of 14 mm and 36 mm, respectively (Fig 5).

Both brooding and non-brooding individuals had a positive and significant relationship between female dry tissue weight and shell length (R^2^ = 0.75, p = 0.001 and R^2^ = 0.81, p = 0.001 respectively, Fig 5). There was no positive relationship between female size and the number of capsules per brood, based on a whole year of sampling (2003–2004, Fig 6 year round). Notwithstanding the above, significant but weak relationships were identified during June 2003, Fig 6).

**Fig 6:**
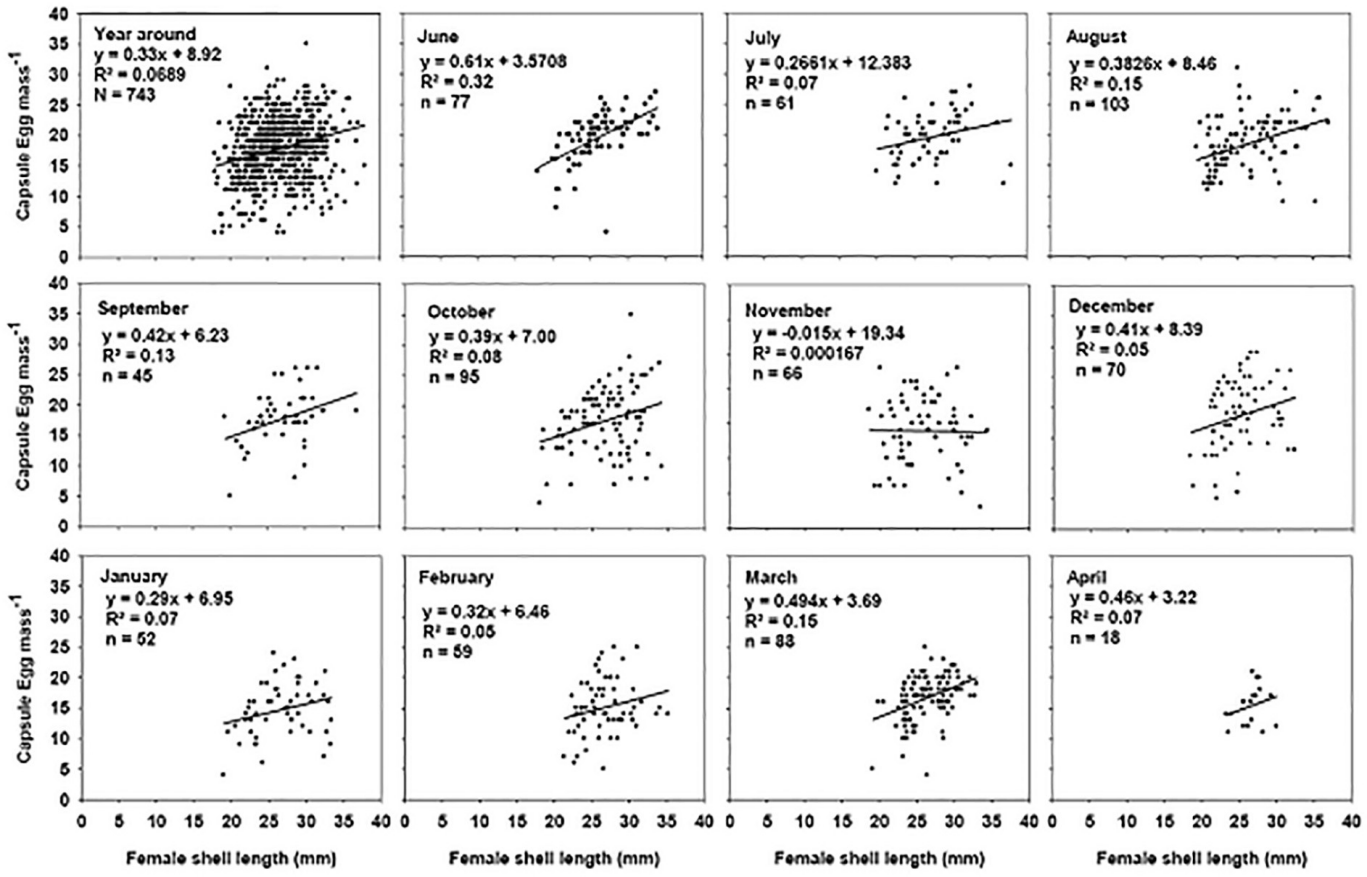
Crepipatella dilatata. Relationship between the size of the female and the number of capsules brooded for samples made during 2003–2004 (yearly, first graph, and for each month independently). May 2003 is not included because of the low number of available data. Very early egg masses were eliminated (stage 1) from graphs in order to avoid including females that could have been in the process of laying at the time of sampling, which could cause us to underestimate the number of capsules in the egg mass. Total N = 743.

**Fig 7:**
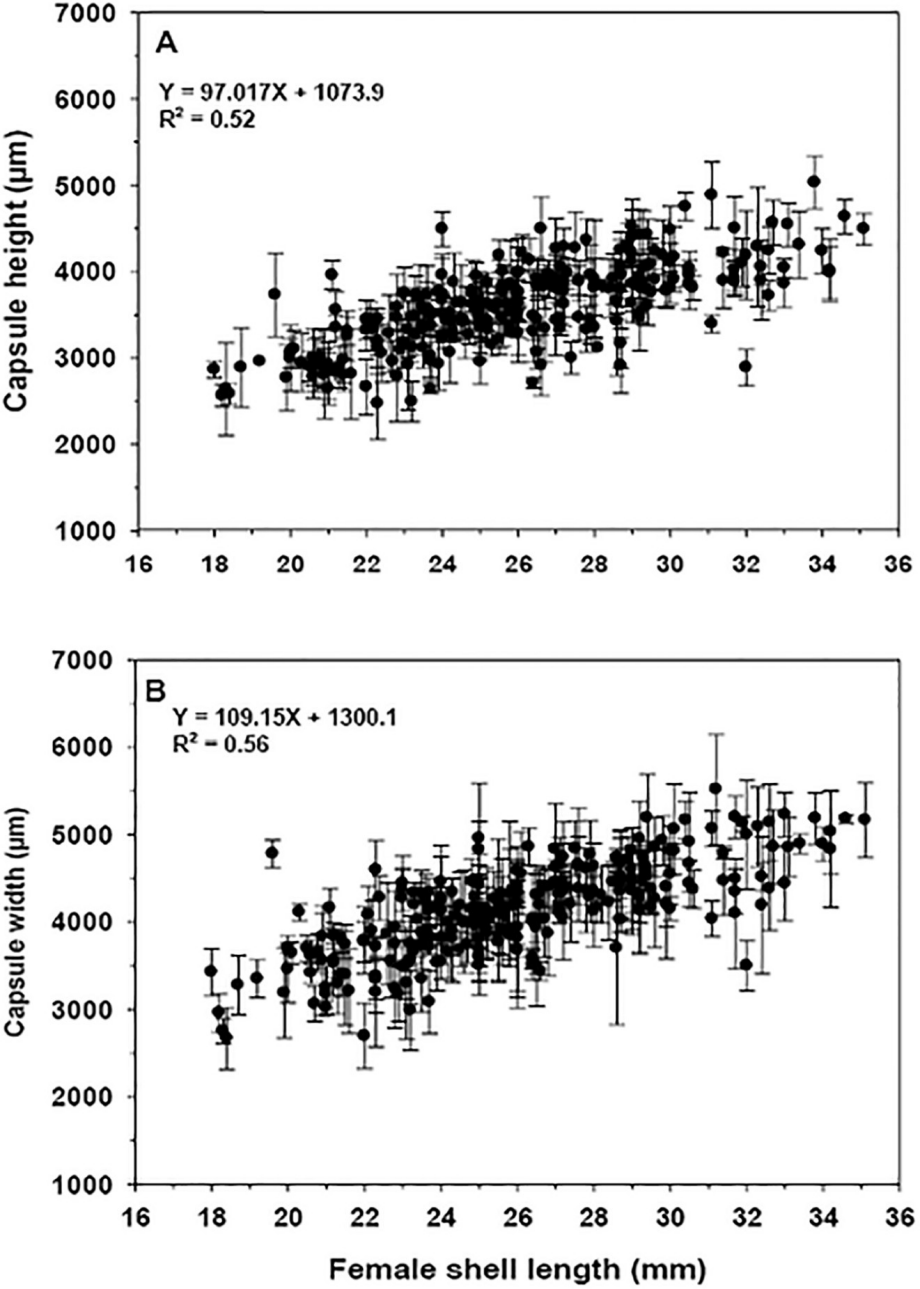
Crepipatella dilatata. Relationship between female size and the characteristics of A) high (N = 267) and B) wide (N = 267) of the brooded capsules. Each point corresponds to the mean of 3 to 5 capsules examined per egg mass).

The regression analysis of all data grouped from the different sampling seasons indicates that the size of the brooders presented a positive and significant relationship with egg capsule height (R^2^ = 0.52, p = 0.001) and width (R^2^ = 0.56, p = 0.001) (Fig 7A and 7B).

Mean capsule height and width did not vary significantly between the different seasons of the year (ANCOVA, F_(3,258)_: 0.551, p = 0.647 and ANCOVA, F_(3,258)_: 0.922, p = 0.430, respectively). Average values of height and width for capsules considering the four seasons of the year were 3597.2 (± 482) μm and 4135.9 (± 520) μm, respectively (Fig 7A and 7B).

### Capsular content and fecundity in *C*. *dilatata*

**Fig 8:**
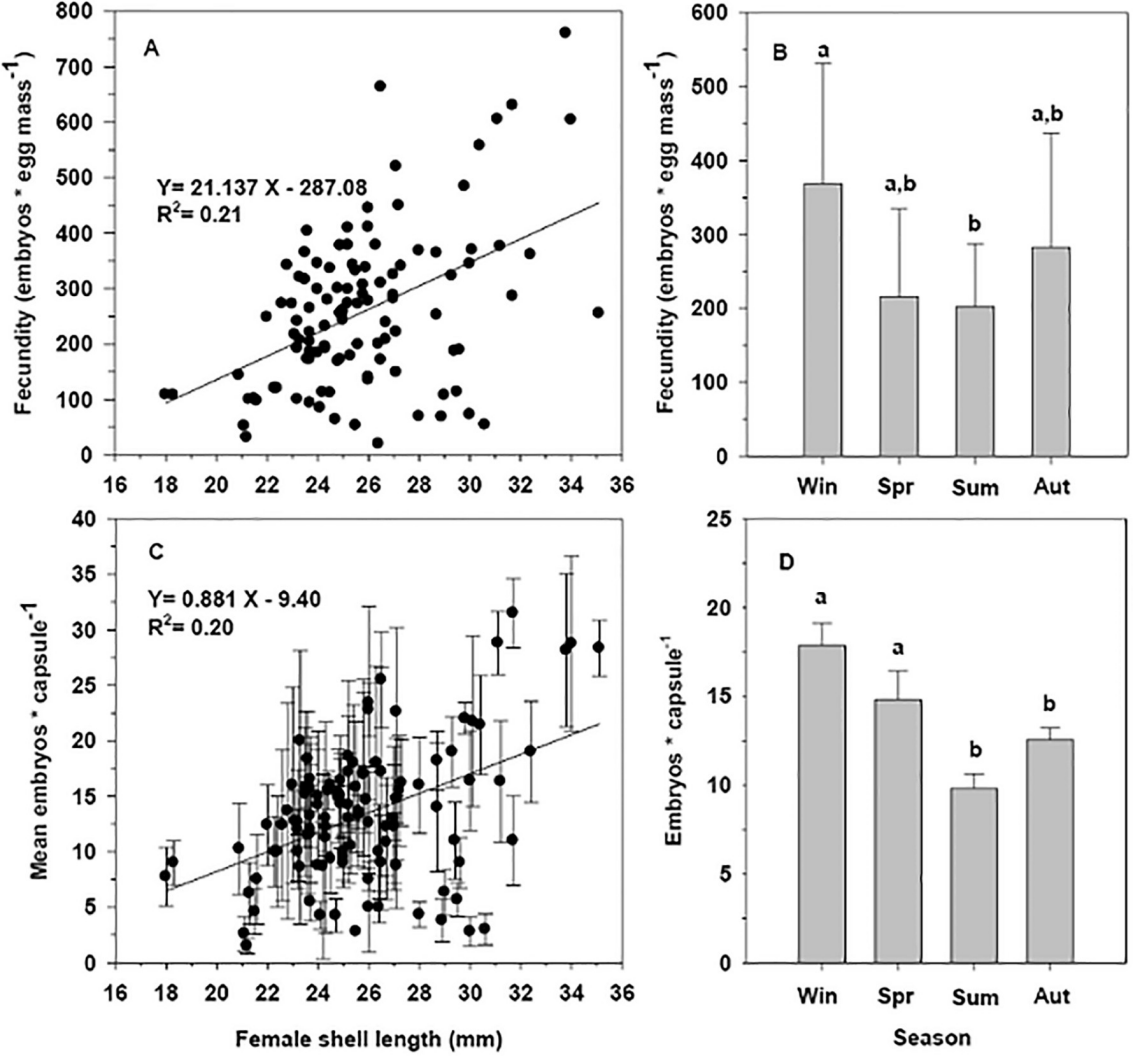
Crepipatella dilatata. Relationship between the size of the brooding females and the number of embryos by A) capsule egg mass (data from all seasons of the year pooled), B) according to the each independent season of the year, C) embryos per capsule(all seasons of the year) and D) embryos per capsule according to each season of the year (N = 108). Data are for all seasons during the period of 2003–2004.

The number of embryos per egg mass of *C*. *dilatata* was significantly—although weakly—correlated with female size (R^2^ = 0.21, p = 0.0001); this analysis included the grouped information for all the seasons of the 2003–2004 sampling period, excluding capsules containing egg stage embryos (Fig 8A). The minimum fecundity recorded for a reproductive event was 20 offspring for a female of 26.4 mm in shell length, while the maximum of 760 offspring was found for a female of 33.8 mm in shell length.

On the other hand, comparisons between the seasons of the year indicate the existence of significant differences in fecundity of females (ANCOVA: F_(3,100)_: 8.3441, p = 0.0003, Fig 8B). Correlations between the number of embryos per capsule and the length of the brooding female, indicate that there was also a weak positive but significant relationship (R^2^ = 0.20, p < 0.0001) (Fig 8C). The number of embryos per capsule differed significantly during different seasons of the year (ANCOVA: F_(3,100)_: 3,075; p = 0.031, Fig 8D). On average, during the winter season, the highest number of embryos per capsule recorded was 18 ± 4 SD, which was 17.4, 45.2 and 30.4% higher than were recorded in the spring, summer and autumn seasons, respectively (Fig 8D).

**Fig 9:**
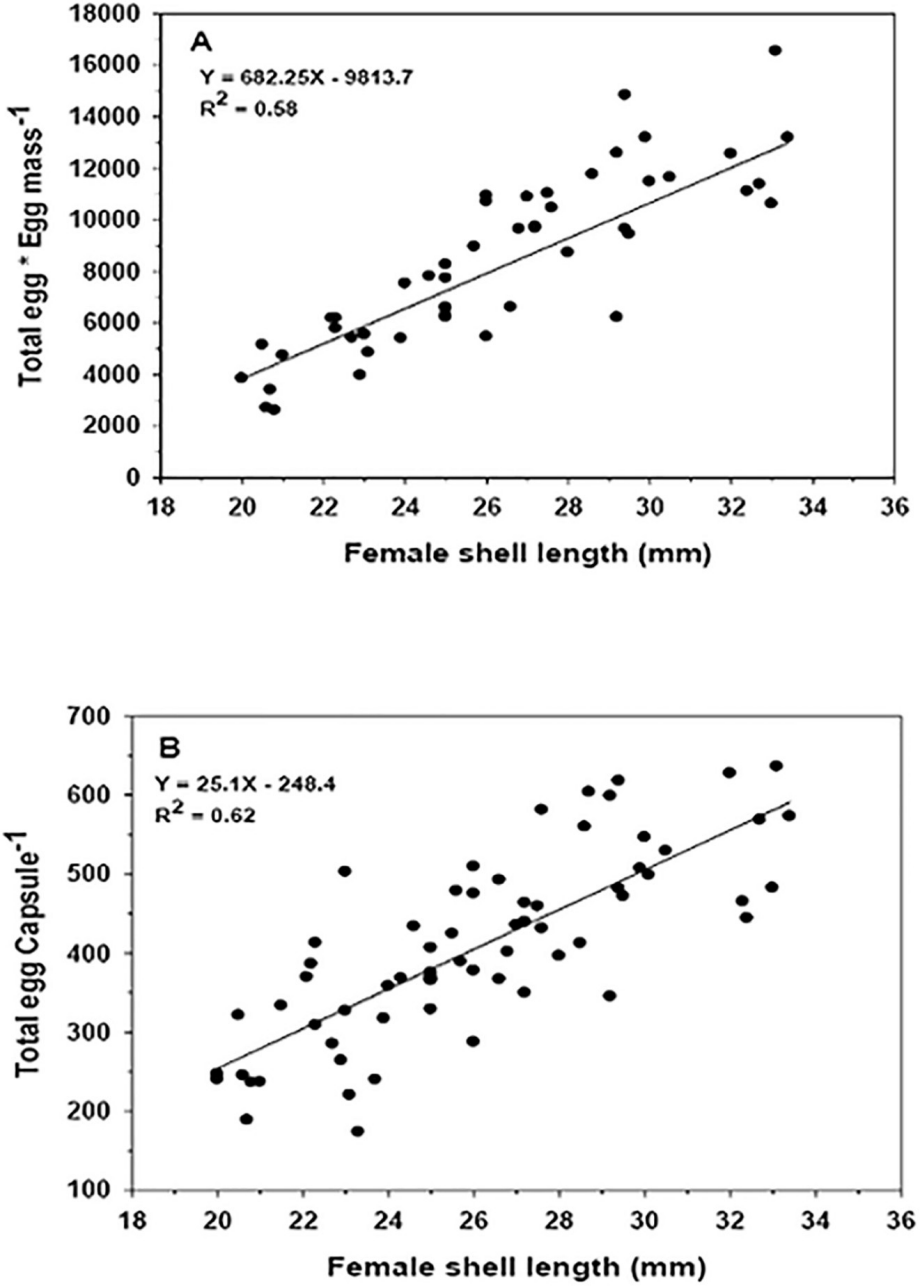
Crepipatella dilatata. Total eggs in the A) capsule egg mass (N = 66) and in the B) individual capsules (N = 66) according to the size of the brooding females. Seasonal data were pooled. Data from capsules containing early embryos (stage 1) were eliminated to avoid potential cases of females in the process of laying egg masses at the time of sampling.

The relationship between the number of total eggs per spawning mass (Fig 9A, R^2^ = 0.58, p < 0.0001) and per capsule (Fig 9B, R^2^ = 0.62, p < 0.0001) as a function of the length of the female’s shell, presented a positive and highly significant correlation over the course of the year.

The lowest number of total eggs recorded for a brood was 2,662 for a female with a shell length of 21 mm. In contrast, the maximum number of eggs identified in an egg mass was 16,536 for a female with a shell length of 33.1 mm (Fig 9A). On average, the number of total eggs (embryos + nurse eggs) per laid egg mass was 8,024 ± 3,227 (mean ± SD). The maximum and minimum number of eggs per capsule was 627 and 173 for females of 32 and 23 mm shell length, respectively (Fig 9B). The analyses indicate that there were no significant differences between the total number of eggs deposited inside an egg mass (ANCOVA, F_(3,61)_ = 2.401, p = 0.076) and inside an individual egg capsule (ANCOVA, F_(3,61)_ = 1.383, p = 0.256), depending on the different seasons of the year.

**Fig 10:**
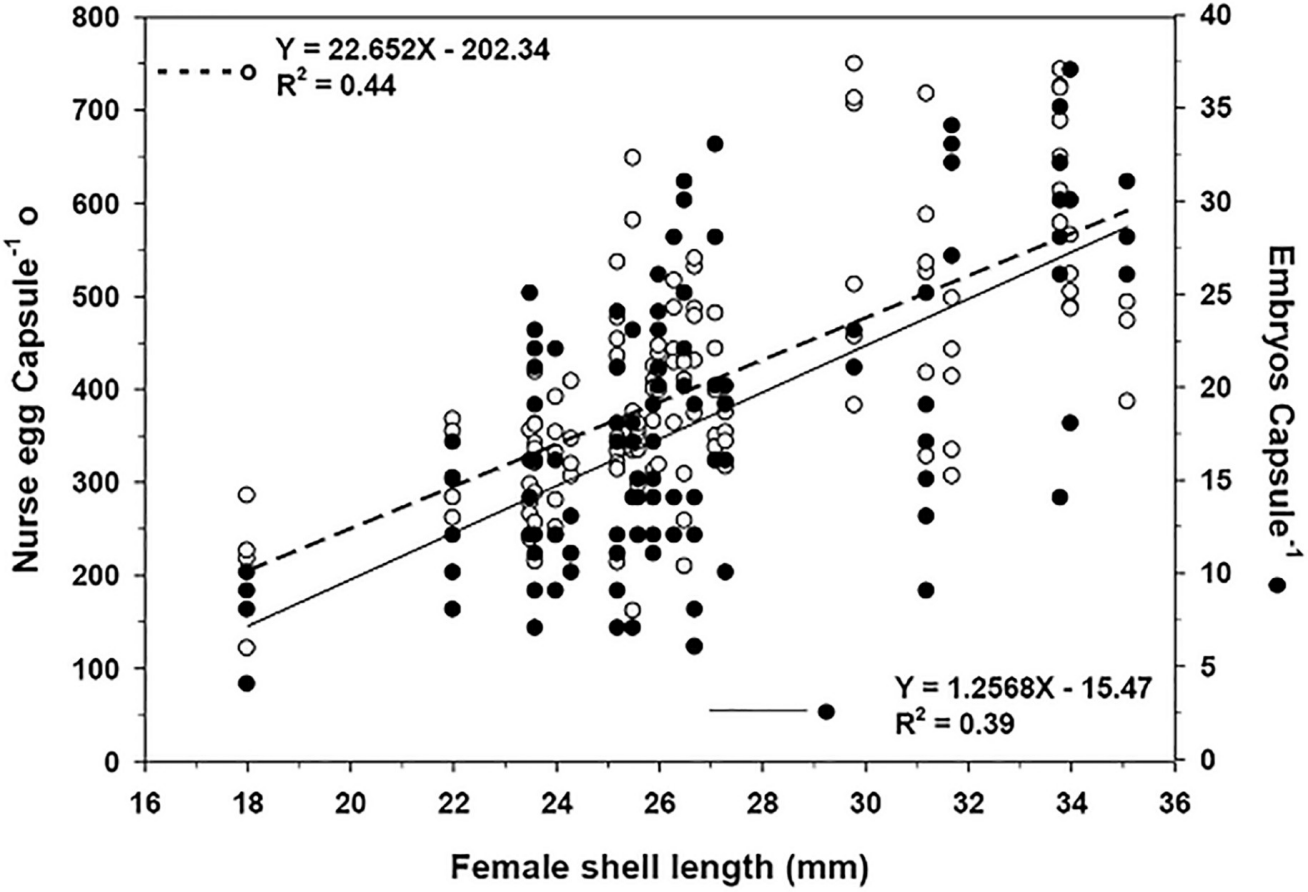
Crepipatella dilatata. Shell length of the brooding female and the characteristics of capsule contents: embryos per capsule (straight line, N = 116) and nurse eggs (dashed line, N = 125) per capsule. In the figure, only information obtained during July 2004 was used in consideration of the specific level of development of the embryos.

The number of nurse eggs contained within an egg mass (before any of the nurse eggs were ingested) during the winter season (July 2004) was positively related (R^2^ = 0.44, p < 0.005) with the size of the brooding female (Fig 10).

The average number of nurse eggs was 212 ± 67 (mean ± SD) for a female with a shell length of 18 mm, a value that increases significantly with increased female size, reaching an average of 674 ± 62 (mean ± SD) nurse eggs for a female of 34 mm shell length (Fig 10). On the other hand, the number of embryos per capsule was also positively related with female size (R^2^ = 0.39, p < 0.005). On average, a female of 18 mm in shell length had 8 ± 3 (mean ± SD) embryos per capsule, which can be increased in the larger females (35.1 mm shell length) reaching 28 ± 3 (mean ± SD) embryos per capsule (Fig 10).

**Fig 11:**
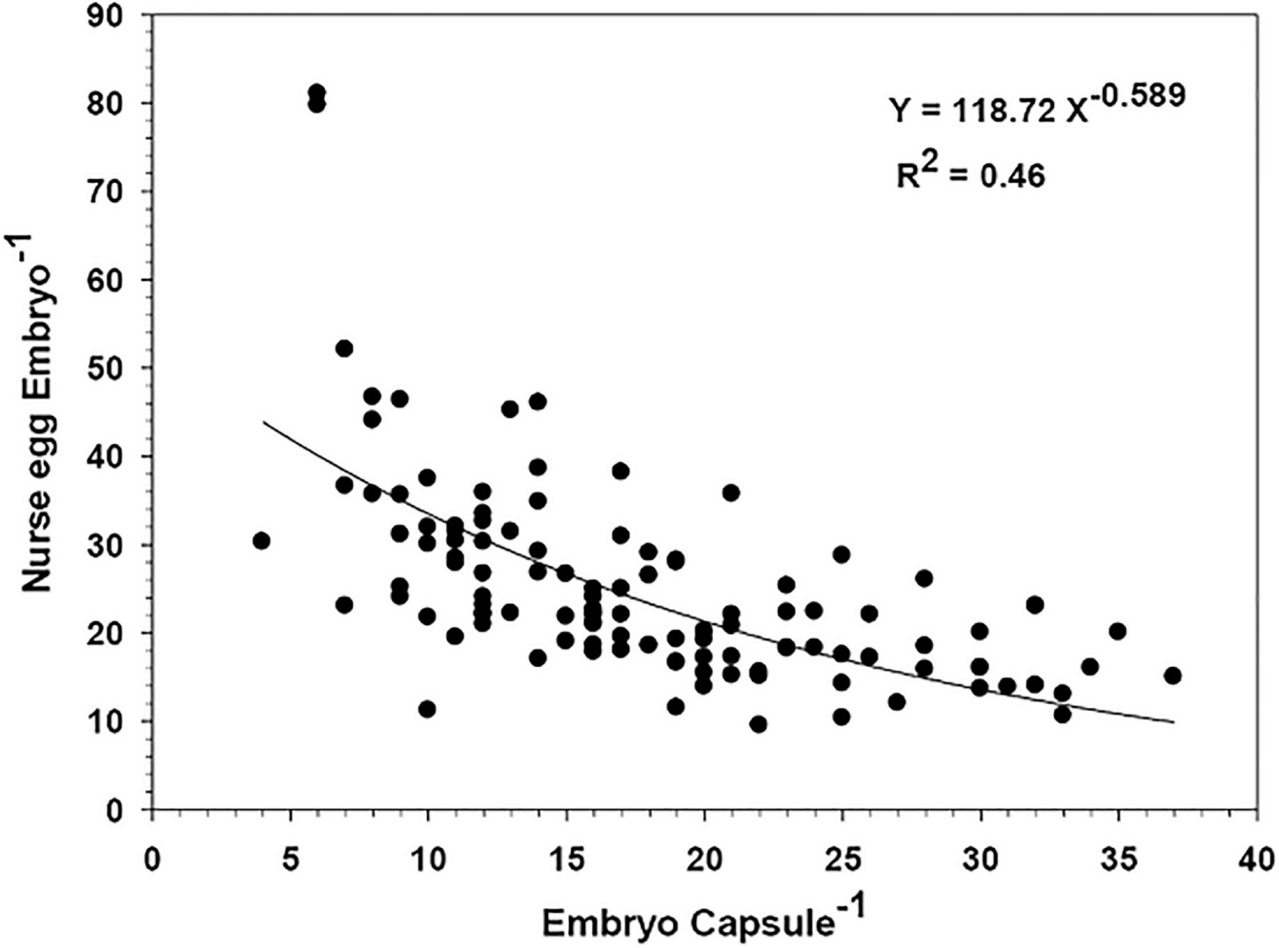
Crepipatella dilatata. Availability of nurse eggs per embryo related to the number of embryos per capsule (N = 114). Sampling date: July 2004.

There was a significant inverse relationship between the number of embryos per capsule and the number of nurse eggs available for each embryo inside the capsule (R^2^ = 0.46, p < 0.0001, Fig 11).

In capsules with approximately 8 embryos, the number of nurse eggs available for each embryo was 32 ± 8, whereas there were about 50% fewer nurse eggs in capsules containing between 35 and 40 embryos (Fig 11).

### At hatching: Juvenile—Veliger ratios

**Fig 12:**
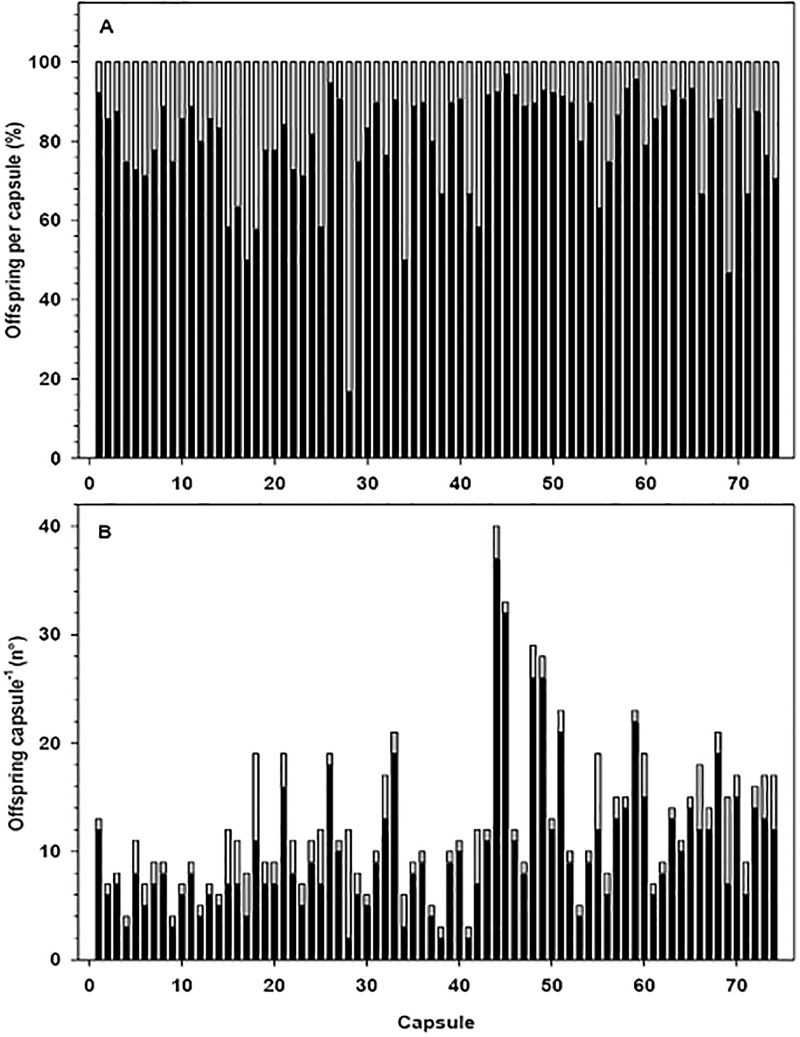
Crepipatella dilatata. Conditions of the offspring in capsules in the process of hatching, in cases in which both veliger larvae (gray bars) and juveniles (black bars) were present. A) Percentage of metamorphosed juveniles and veliger larvae (not metamorphosed), B) number of offspring per egg capsule. Each bar represents data from a particular capsule. N = 74 capsules from 33 egg masses.

Thirty-three of the 51 egg masses examined presented at least one hatched capsule where both veligers and juveniles were present. The rest of the egg masses that were examined at hatching contained only juveniles. Seventy-five percent of the 293 capsules examined from the 51 egg masses contained only metamorphosed juveniles at the time of hatching. In the remaining 25% of the capsules, veliger larvae were also found, individuals that had not yet completed the process of metamorphosis. There was no significant relationship between female size and the developmental condition of embryos at hatching (P > 0.05). Considering only those capsules containing both juveniles and larvae, it was observed that on average they hatched when 79% of the offspring inside were metamorphosed (i.e., were juveniles) (Fig 12A). In more extreme cases, hatching occurred when only 18% of the encapsulated individuals had metamorphosed (Fig 12A). These minimum values corresponded to only 2–4 individuals metamorphosed inside the hatching capsules (Fig 12B).

**Fig 13:**
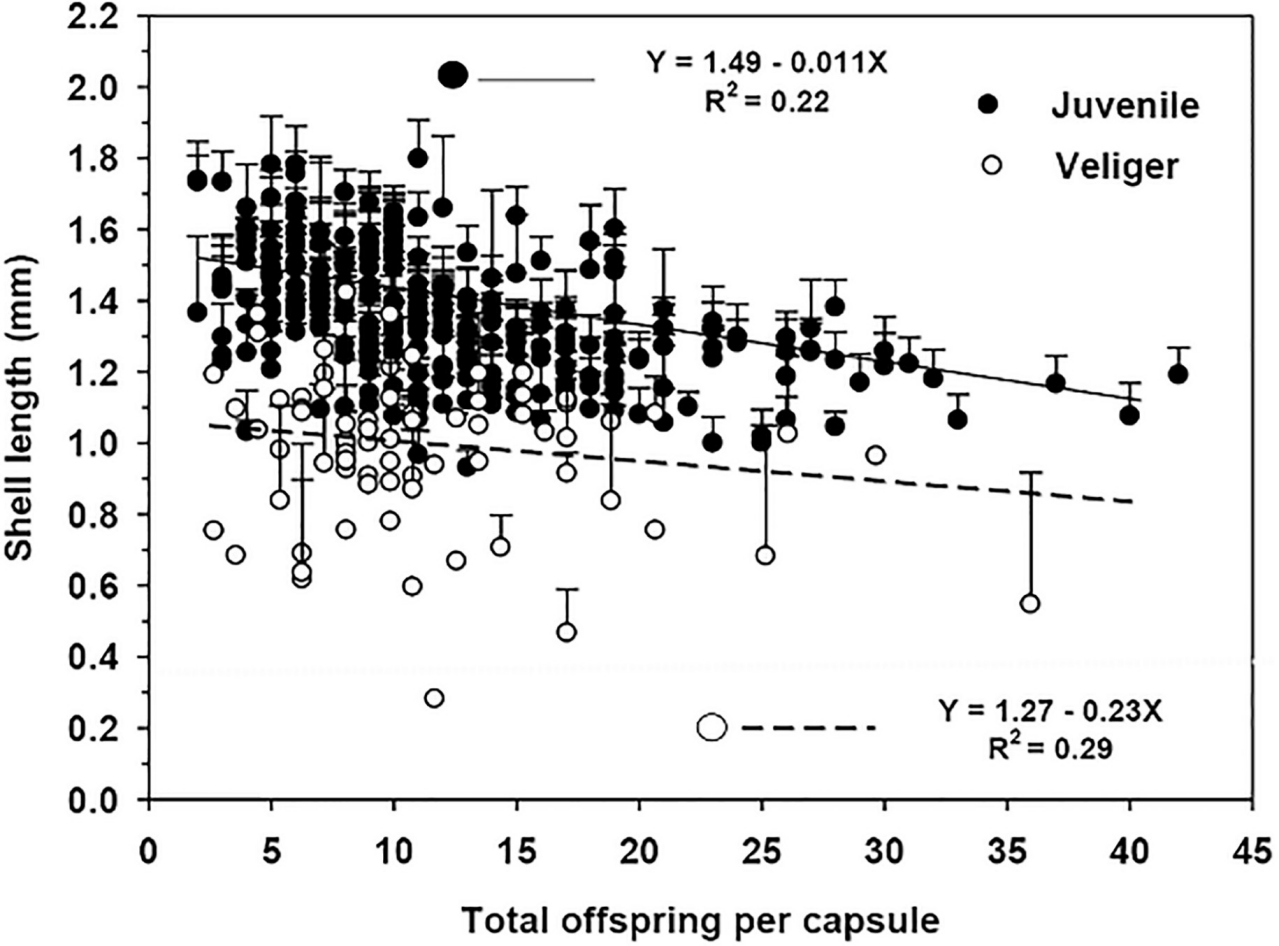
Crepipatella dilatata. Average shell lengths (± SD) of the offspring (juveniles and veliger larvae) at the time of hatching according to the number of siblings with which they had to share the availability of nutritional eggs deposited by the mother at the time of laying. Figure includes data from capsules containing only juveniles and those containing both veligers and juveniles at the time of hatching. Experimental date: December 2013.

In turn, the hatching juvenile would have a size greater than 1 mm in shell length. In contrast, veligers that were present in the capsules at the time of hatching had an average shell size significantly lower than that recorded for their already-metamorphosed siblings (Fig 13).

The size of juveniles at hatching was inversely related to the number of capsule mates (Fig 13).

## Discussion

Individuals of *C*. *dilatata* changed sex from male to female when individuals reached a shell length of approximately 20 mm. In the studied population, brooding females were identified almost year around, with exception of two months, where less than 3% of individuals were found to be brooding. Our results indicate that there is a strong relationship between the size of the brooder, the capsular dimensions, and embryonic content. Interestingly, most individuals brooded twice a year, but in exceptional cases, some females were able to brood up to 5 times a year.

### Brooding females, sex change / minimum size of incubation

Females of *C*. *dilatata* from the Quempillén estuary population brood during almost the entire year, with a short resting period during May and June (Fig 1B). This short period of resting occurs when the minimum values of temperature, low mean salinity, and poor food concentrations in the water column of the Quempillén estuary had been recorded [9,33]. These environmental factors can cause high levels of oxidative damage, impacting the reproductive process, as was corroborated experimentally for *C*. *dilatata* [47]. Temperature as a determining environmental factor also plays a key role since it controls and synchronizes the reproductive processes in many groups of marine invertebrates, stimulating gametic production through the synthesis of hormones [48]. For this species, previous studies have also indicated the existence of an extended reproductive period throughout the year [49]. In *Crepipatella peruviana*, a sympatric calyptraeid species, close to 80% of the females of a population of Chiloé Island (Southern Chile) have been also been found to be brooding during summer [50]. In the case of the invasive species *Crepidula fornicata* in the UK, the maximum levels of brooding females sampled during the summer reached between 78% and 92% [51].

*C*. *dilatata* is a protandric hermaphrodite species, a characteristic common to species of the family Calyptraeidae [15]. In previous studies, the sex change in this group of gastropods has been identified by direct observation of the presence / absence of penis or genital papillae as an indicator of the sexual condition of the specimens sampled (*e*.*g*. *C*. *fornicata*, Bohn *et al*. (2012) [51]). According to our gonadal histological studies, individuals were approximately 20 mm in shell length when the sex change occurred in the sampled population of *C*. *dilatata* in the Quempillén estuary. This change was corroborated by the identification of the minimum size of females that brooded egg masses. Individuals of this population have a functional male gonad when the shell length is near 8 mm, and most individuals function as males when between about 7.5 mm and 19.0 mm in shell length (Fig 2) [34].

The size at which sex change occurs in this group seems to be species-specific [52]. In *C*. *dilatata*, sex transition seems to be a fast process, since previous histological studies have shown that sex change occurs in individuals between 11.5–19.9 mm in length and was identified only in about 8% of the adults [34]. The observations of Gallardo (1996) [49] on *C*. *philippiana* indicate that sex change in that species would also be rapid. Considering the above, Richard *et al*. (2006 [53]) identified in *C*. *fornicata* a very low proportion of individuals in an intermediate state of transition, which would indicate that in this species, the fugacity of this sexual transition and the speed with which this process takes place.

On the other hand, the minimum brooding size recorded in this study (≈20 mm shell length) does not differ substantively from the information previously given for *C*. *dilatata*, which indicates the existence of female gonadal activity or presence of brooded egg masses in the smaller individuals with shell lengths of 17.3 mm [34], 18 mm [29] or 20 mm [25]. However, Zelaya *et al*. (2012) [54] indicate the existence of brooding females from 12.7 mm shell length, in a subtidal population of *C*. *dilatata*.

### Extension of the reproductive process

*C*. *dilatata* is a species that broodings practically throughout the year. Two other calyptraeid gastropods in Chile (Southern Hemisphere), *C*. *peruviana* and *C*. *philippiana*, living in similar latitudes to those of *C*. *dilatata*, also reproduce almost continuously throughout the year [49]. Information on calyptraeids from the Northern Hemisphere indicate that reproductive processes are also extended in the year. For example, for *Crepidula fornicata* living along the east coast of USA, females started breeding in May and continued breeding until September [55]. Along the European coasts, this invasive species was found to brood egg capsules from March to September and larvae were hatching along the U.K. coast even during the period of lowest water temperatures (< 7 °C) [51], at which they continue to develop. A previous report indicates that this invasive species reproduces between February and September in the waters along the French coast, reaching its maximum peak during May-June, when 80% of the females having been found to be brooding embryos [53]. In *C*. *fornicata* the presence of high concentrations of chlorophyll and a higher water temperature encourage the reproductive process, particularly favoring the start of the incubation period earlier in the year [56]. In the calyptraeid species with direct development, such as *C*. *adunca*, reproduction has also been recorded throughout the year [4] (Table 1). Notwithstanding the foregoing, more seasonal reproduction has been described in other calyptraeids, such as in the mixed development species *C*. *lingulata*, where reproduction has been identified only during the summer season [4] or in *C*. *convexa* where incubation would be between April and August for a population in Delaware [57] or between May and September for an estuarine population in Connecticut [58] (Table 1).

**Table 1:** Brooding periods in different species of Calyptraeids from both Hemispheres.

Specie	Brooding period	Location	Author
*C*. *dilatata*	10 months per year	Southern Hemisphere	This research
*C*. *peruviana*	Mostly year around	Southern Hemisphere	[25] [50]
*C*. *philippiana*	Mostly year around	Southern Hemisphere	[49]
*C*. *fornicata*	March–September	Northern Hemisphere	[51]
*C*. *fornicata*	February- September	Northern Hemisphere	[53]
*C*. *adunca*	Year around	Northern Hemisphere	[4]
*C*. *lingulata*	Summer season	Northern Hemisphere	[4]

### Events of laid egg mass per year

Females from the Quempillén population presented between 1 and 5 events of egg mass deposition per year, with a mode of 2 reproductive events. This high reproductive frequency in *C*. *dilatata* seems to indicate that during brooding, a gametogenesis process would be acting in parallel in the development of a new generation of gametes. A situation like this has been identified for the sympatric species *C*. *peruviana*, a species in which brooding females have been found to simultaneously have a gonadal development with the presence of several cohorts in gametic development [59], implying that a new egg mass laying event would occur very soon after the hatching of the offspring. Multiple annual reproductive events for calyptraeid species in Chile have also been identified in *Trochita calyptraeformis* (previously *Calyptraea (Trochita) trochiformes*, see Collin (2003b) [30], a species that can brood up to 5 or 6 times per year [60]. On the other hand, females of *C*. *peruviana* have been found to have up to 7 reproductive events throughout the year, while the reproductive mode of the population was identified in 4 annual ovipositions [50]. In the invasive limpet *C*. *fornicata*, which exhibits mixed development, multiple spawnings per reproductive season have also been identified, typically averaging between 2.2 and 2.9 reproductive events per year along the UK coast [51], and between 2 and 4 ovipositions per female in populations from the French coast [53]. Similar results were observed for *C*. *lingulata*, for which at least 3 broods per female have been identified during the summer season [4].

Females in our study showed no significant reduction in tissue content during the egg mass laying process. This is consistent with the results of Chaparro *et al*. (1999) [29] for the same species, where no differences were found in the tissue content between brooding and non-brooding individuals. This suggests that the reproductive output during each event for *C*. *dilatata* is not so high with respect to the biomass content of the females, so that the egg laying does not substantially decrease the tissue content of the females during the process of oviposition.

### Females size and capsule characteristics

In general, in *C*. *dilatata*, the size of the female does not determine the number of brooded capsules. However, monthly analyses of female shell length and the number of brooded capsules (Fig 6), identify one month (e.g. June) in which this relationship is weak, but significant, as had been recorded previously by [29] for the same species. These positive relationships occur at the beginning of the breeding cycle and coincide with periods of low temperatures in the estuary. However, female size is a major determinant of the characteristics of the laid capsules. Thus, the mean height and the width of the capsules (and therefore the capsular area) increased significantly with the size of the breeding female (Fig 7), a situation similar to that identified for *C*. *dilatata* in the South of Chile [29]. In the case of *C*. *philippiana*, there was no positive relationship between female size and the size of the capsules, although the number of capsules laid per female was related to the size of the reproductive adult [49], the opposite of what we recorded for *C*. *dilatata* in the present study. In *C*. *dilatata*, the fact that some females deposited few egg capsules did not imply alterations in the relationship between the size of the female and the capsular dimensions.

### Nurse eggs and fecundity

Larger capsular dimensions correlate with the increase in the total number of eggs (nurse eggs + embryonic eggs), both within individual egg capsules and for the entire egg mass (Fig 9). In this context, there is a weak increase in the number of embryos per capsules and per egg mass with increasing female size. In some seasons of the year, this relationship was not significant.

The fecundity of *C*. *dilatata* is low in comparison to that of most other calyptraeids that have been studied, considering that the biggest females generated a maximum of 800 juveniles per egg mass (Fig 8). The available information indicates that from the initial number of eggs laid inside the capsules, only approximately 5% develop as embryos. Previous studies have indicated that in *C*. *dilatata*, between 3 and 8% [22], or 5 to 6% [25] of the initial encapsulated eggs continue their development as embryos, using nurse eggs as an energy source during their encapsulated development [42]. This fecundity is higher than that recorded by species such as *C*. *philippiana* that only generates one juvenile per capsule [49], but well below those species of Calyptraeids with mixed development, where the production of larvae can reach levels close to 10,000–15,000 per brood in *C*. *fornicata* [53] or *C*. *peruviana* with an average of 34,000 larvae per female [61]. The specific analysis of egg masses from the end of winter season (July) showed a clear relationship between female size and the embryos number present inside the capsules. This was also true for the number of nurse eggs per capsule, a situation implying that the capsules produced by larger females also increase the availability of nurse eggs, but not in the same proportion in which the embryos per capsule increase.

In this species, the ingestion of nurse eggs begins once embryos have developed the larval velum and its ciliature [62]. Each nurse egg is ingested whole, after being rotated by the embryos using the ciliary crown of the velum. During the rotation process, the nurse eggs seem to diminish their external rigidity favoring the spherical shape change to a more elongated one at the moment when the ciliature of the buccal region begins the suction / intake of the egg towards the inside of the esophagus [26]. It takes a fair amount of time before all of the nutritional nurse eggs within each capsule are consumed. Nurse eggs are consumed during most of the encapsulated period, including by very well developed veligers. The amount of nurse eggs in the capsules decreases reaching values close to 0 (zero), moment when the offspring is complete developed, close to metamorphosis and hatching stages.

The mechanism of ingestion of the nurse eggs had not been clearly established for most of the calyptraeids, whereas for other species of gastropods (e.g., the muricid *Acanthina monodon)*, ingestion is carried out when the trocophore stage is reached, before the velar ciliature has developed; nurse egg intake occurs in a short period of time in those species [63]. In the same context, the ingestion of nurse eggs by the gastropod *Searlesia dira* also occurs in the trocophore stage using the dense circumoral ciliature [64].

### Hatching

In those capsules that were in in the process of hatching, the mean size of the hatchling was larger than 1 mm in shell length. These sizes (1075–1600 μm SL) are within the range) of those previously reported by Chaparro & Paschke (1990) [62] for the same species. Gallardo [22] indicated that hatchings were between 900 and 1300 μm in shell length, although in populations from south-austral of Chile, the hatchlings can be up to 1.6 mm in shell length. During the hatching experiments, we recorded in some capsules both metamorphosed juveniles and veliger stages (Fig 12). The latter had shell lengths that were < 1000 mm, smaller sizes than the juvenile capsule-mates. These differences in the size and level of development during hatching, between the offspring from the same capsule, suggest intracapsular competition for food between capsule-mates. Our results indicated that juvenile hatching sizes were also influenced by the number of sibling embryos that shared the same capsule. Thus, the largest size of hatchling occurred in capsules containing the lowest densities of capsule-mates, possibly because the nurse eggs were distributed among a smaller number of siblings during encapsulated development. The above is similar to what was identified for *Searlesia dira*, where the number of nurse eggs that an embryo consumed was inversely proportional to the number of sibling embryos inside each capsule; this impacted on a smaller size of hatching [64]. In 75% of the capsules monitored for *C*. *dilatata*, all offspring had metamorphosed into juveniles by the time of hatching. In cases where there were still veliger larvae within the capsules at hatching, commonly more than 79% of the offspring were already metamorphosed. In a few cases, the hatching occurred when the capsules contained only 2–4 juveniles; hatching nevertheless took place, despite the low number of juveniles. In our studies, hatching occurred in the absence of the mother, corroborating that in this species the presence of the mother is not essential for the capsular opening, contrary to what occurs in the calyptraeid *Crepidula navicella*, for which the hatching of juveniles from the capsule depends on the presence of the brooding female [65]. Although in *C*. *dilatata* hatching can occur without female presence, it is possible that the female could play a role in aspects of the process, such as synchronization of hatching, allowing the juveniles to exit in a limited period of time [66]. A greater hatching synchronization in the presence of the female has also been described in other species of brooding invertebrates [67,68].

On the other hand, comparisons of hatching sizes among calyptraeid species make it possible to assume that adelphophagy somehow results in a greater variance in the sizes among hatching individuals [69]. This greater dispersion in the sizes of hatching is evidenced in *C*. *dilatata*, considering the range of between 0.70 and 1.79 mm (including sizes of veliger and juveniles inside the capsules at the time of hatching) as extreme values in all the individuals controlled during the present research. The hatching sizes previously reported for this species were usually based on the sizes of the offspring found under the maternal shell around the capsules. Those juveniles were found when researchers detached the females from the substrate. These values could not consider the exact moment of hatching, or even the time in which some of the first offspring left the capsules but remained beneath the female’s shell. The influence of extra-embryonic food (including the consumption of dead siblings inside the capsule) has been tested experimentally in a number of different calyptraeid species such as *Crepipatella peruviana* [28], *Bostrycapulus calyptraeformis*, *Crucibulum spinosum*, and *Crepidula cf*. *marginalis* [70]. In these species, dead congeners were offered as an extraembryonic source of food for the siblings that continued their development inside the capsules. Particularly, in *C*. *peruviana*, larger sizes were reached as well as a greater dispersion in the sizes of the hatched larvae (species with mixed development) and in the last two species, an increase in shell length and in the diameter of the velum of the embryos that fed on the dead siblings inside the capsules. The importance of the extraembryonic food inside the capsules in calyptraeids is reflected in the results of the largest variation in the size of the offspring for *C*. *peruviana* [28], which would respond to a differentiated use of the extra embryonic food represented by their dead congeners inside the capsules for *C*. *navicella* (former *C*. *cf*. *onyx*, [65]), on the other hand, this would be due to different numbers of nurse eggs ingested by embryos within each capsule, or perhaps to the different distribution of the nurse eggs within the capsules [69].

According to the results of this investigation, it is evident that *C*. *dilatata* has a high reproductive potential, considering the large number of months that individuals are actively reproducing, and the number of brooding events per female each year, as well as the use of brooding during the reproductiveprocess, which protects the offspring until they metamorphose into juveniles that are capable of going outside with autonomous feeding and movement capabilities [71]. The brooding mode of reproduction protects the embryos against environmental stressors typical of medium-high latitudes and especially in frequently changing environments such as estuaries [72,73]. The extended reproductive period during the year that was documented in this study could explain the variations that are identified over time as part of the reproductive process, *e*.*g*. different maternal reproductive output depending on the season of the year when this process is carried out.

In this research we reported several issues related to *C*. *dilatata* reproduction. However, there are several other issues that need to be studied in the future, for example the extent of female participation in the hatching process, the mechanisms involved in sex change, as well as the factors enabling all year reproduction and the potential strategies involved in the distribution of seasonal energy expenditures within the capsules comprising the egg mass.

## Supporting information

S1 Dataset Reproductive measurements for *Crepipatella dilatata* individuals. (XLSX)
